# Upon Filtration of the Air in Connection with Fermentation and Putrefaction

**Published:** 1854-06

**Authors:** H. Schröder, Th. Von Dusch


*Upon Filtration of the air in connection with Fermentation and Putrefaction.* By H. SCHRÖDER, and Dr. TH. VON DUSCH.

(Translated for the Examiner.)

§1. In the year 1837, Dr. Schwann, of Berlin, communicated a series of experiments, which proved that putrefaction did not occur in a freshly boiled infusion of flesh, and that the vinous fermentation did not take place in a freshly boiled fluid, previously susceptible to fermentation, if the air that was suffered to enter had been first exposed to a red heat. It was proved by these experiments, that it is not the oxygen, at least not it alone, that causes the vinous fermentation, the putrefaction of an infusion of flesh, or even the formation of mould and infusoria, but a substance contained in the atmosphere and destroyed by heat. Schwann considered it probable that fermentation and putrefaction were induced by sporules of microscopical cryptogami and infusoria, contained in the air, which developed themselves and increased at the expense of the fermenting or putrefying substance, and thus continued the process. These sporules or germs existing in the air, are, however, destroyed by heat. Similar experiments with like results were made by Ure and Helmholz.

§2. Regaud de l'Isle has shown in his examination of the miasmatic influences of the Pontine marshes, that an interposing wood is a protection from the noxious effects,* and Becquerel says, "Une forét interposeé sur le passage d'un courant d'air humide, chargé de miasmes pestilentiels, prèserve quelquefois de ses effets tout ce qui est derriére elle, tandis que la partie découverte est exposeé aux maladies. Les arbres tamisent donc l'air infecté, et l'epinent en lui énvelant ses miasmes."†

Finally, Löwel communicated last year a series of remarkable experiments, upon the crystallization of an oversaturated solution of sulphate of soda, and proved that such a solution, which, when in contact with air, crystallises almost instantaneously, does not crystallise if the air has been previously filtered through a layer of cotton.

§3. The facts above mentioned, being all that is known with

*Bibl. Univers. vol. xiii.

†Compt. Rend. hebd. xxxvi. 12.

regard to fermentation and putrefaction, in connection with the filtration of air, led one of us in January, 1853, to the supposition, that a freshly boiled organic substance, in contact only with air properly filtered, would be protected from fermentation or decomposition. In order to test this supposition, we performed the following experiments.

§4. Cotton was selected as a means of filtration, because it is known that it retains upon its surface infectious diseases, and is even capable of conveying them to a distance.

The apparatus used for filtering the air, consisted of a tube, about 1 inch in diameter and 20 inches in length, filled with raw cotton, moderately compressed; both ends were closed with waxed corks, through one of which was passed a short, open glass tube about ¼ inch in diameter; the other end was connected by a tube of similar diameter bent at right angles, with the flask containing the meat or infusion to be experimented upon. The flask itself was connected by a tube of the same diameter, with a gasometer or air-tight vessel holding about 1 cubic foot of water, and provided with a discharge cock at the bottom, and another cock to intercept the communication between it and the flask; besides an opening for introducing water, which could be perfectly closed. The joints all being air-tight, it is evident that as the water ran out of the gasometer, fresh air must enter through the cotton and the flask to replace it. When all was in order, the discharge cock was so regulated that the water could escape in drops only, and the air constantly passed through the apparatus in proportionate amount. Before being put into the glass tube, the cotton was heated in a water-bath, and the contents of the flask in all the experiments were brought to ebullition, which was continued until the tubes were heated up to the part where the cotton commenced.

§5. The first experiment was commenced on the 9th of February, 1853. Two flasks, placed side by side, each containing meat, and the freshly boiled decoction thereof, were made use of. One vessel was connected with the filtering apparatus described above, the other was left open. The meat and the decoction in the open flask, began in the second week to develop an intolerable odor of putrefaction, so that it was necessary to remove it from the laboratory.

On the 6th of March, we opened the other flask, through which, during the whole time—that is during 23 days and nights—filtered air had been passed. The appearance of its contents was entirely unchanged. There was no trace of odor, but upon being heated, the pure characteristic smell of fresh unseasoned broth was developed.

§6. The experiment was repeated in a warmer season of the year, 20th of April.

*a.* We placed some meat in water, as described in §4. The treatment was the same as in §5, except that the current of air was only passed through during the day, and the vessel closed at night.

*b.* Beside this, was placed in an open flask, fresh meat boiled in water.

*c.* At the same time, a flask containing similarly prepared meat was closed with a waxed cork, through which was inserted a glass tube about 12 inches in length, and 1 line in diameter, the object being to retard the entrance of air.

*d.* In the fourth flask, we put meat boiled in water, and closed with a loose stopper of cotton, over which a large padding of cotton was placed, fastened to the neck of the flask by a silken thread. Upon the cooling of the flask, the fresh air entering must necessarily be filtered in passing through the cotton.

In the second week, the meat in the open flask (*b.*) underwent stinking putrefaction, and was obliged to be removed from the laboratory.

On the 10th day, a thick growth of mould was observed in the flask with the narrow glass tube, (*c.*) At the expiration of 19 days, upon being opened, only a mouldy smell was perceived, not the odor of putrefied meat.

The two flasks, (*a.* and *d.*) through which filtered air alone had been passed, were opened on the 14th of May, at the expiration of 24 days; no mould formation or any striking change of substance was perceived; a whitish appearance was observed in some parts of the meat, which had not been noticed at least by us before.

The substance in both flasks was found, upon opening, to be without odor; upon being heated, the unchanged smell of fresh broth was developed; the taste was that of fresh unseasoned broth. Like fresh broth, it reacted slightly acid. The distillate of a part of this, was entirely neutral.

By these experiments it is therefore established, *that meat, freshly boiled in water, and freshly boiled broth, remain for several weeks completely unchanged, if only such air as has been previously filtered through cotton is suffered to enter.*

§7. On the 14th of May we took from Grohe's vinegar manufactory of this place, some freshly boiled sweet malt-wort, to which some hops had been added. It smelt and looked like beer wort, tasted sweet, and reacted only slightly acid. This wort was put in the flask connecting with the filtering apparatus, and treated for 23 days, as described in §4. In the last eight days a cubic foot of air was daily drawn rapidly through, so that a visible depression was caused upon the surface of the liquid. An open flask was at the same time placed beside it and filled with freshly boiled wort. After eight days the formation of mould commenced in the open flask; the liquid became also cloudy, whilst that in contact with the filtered air was perfectly clear, and remained free from mould. On the 6th of June, 23 days after, we opened the flask; the liquid was as clear as at the beginning of the experiment, and developed upon being heated the odor of unchanged wort. The taste was sweet and unaltered, and the reaction slightly acid, as before the experiment. Examined with Steinhold's beer test, we obtained 0. p. c. of alcohol, and 7.9 p. c. of extract of malt. We had, previously, neglected to make a determination of the value of the liquor experimented on. We could only, therefore, compare it with a new sample of fresh wort, taken from the same manufactory. This gave with Steinhold's test, 0. p. c. of alcohol, and 7.7 p. c. of extract of malt, therefore of the same value, excepting the slight concentration of the first fluid by evaporation during the experiment. *We believed to have established by these experiments, that at the temperature of May and June of this year, a sweet fermentable wort will remain entirely unchanged for weeks, if only such air has access to it as has been previously filtered through cotton.*

§8. With the new sample of wort above mentioned, we commenced on the 6th of June another experiment. We wished to see, as a farther check upon the experiment, if upon the removal of the cotton from the filtration tube, (the treatment otherwise being the same as in §4,) the contents of the flask would remain *unchanged.* An open flask was again placed beside it. The formation of mould commenced in the latter, within the first week, but not until after 12 days in the one connected with the tubes. It commenced with a rapidly growing speck of mould, exactly on the spot where the current of air came in contact with the surface of the liquid. The liquids in both flasks became cloudy. It was, therefore, evident that protection from these changes could only be found in filtering the air through cotton.

§9. It was now of interest to ascertain, whether under like circumstances, boiled fresh milk would remain unaltered, or whether it would curdle or putrefy. But all the experiments which we made in the months of June and July, gave only *negative* results. The milk coagulated quite as rapidly in filtered, as in open air; and in every case, the odor of putrefying casein was developed, as soon with the former as with the latter. The formation of mould was, however, entirely prevented by the filtration of the air.

This negative result reminded us of a similar one, obtained by one of us, in connection with L. Gmelin, in 1846, in regard to the behaviour of milk, when placed in contact with a large amount of confined air previously heated.

Negative results were also obtained in all experiments with fresh meat that was heated in a water-bath, first being placed in a flask without the addition of water; the flask while yet hot, was closed as in §6 *d.*, with a stopper of cotton, and the neck surrounded by a thick padding of the same material. The meat became offensive, as quickly as in the open air or in a flask, which was corked as in §6. *c.* communicating with the air only through a long narrow tube. The only difference was, that the greenish-brown liquid, that in the open flask surrounded the putrefying pieces of meat, was observed under the microscope to be alive with infusoria, with Monas termo or at other times with Fibrio lineola; whilst in the same liquor that putrefied in filtered air, Fibrio lineola decidedly did not appear, and even Monas termo could not be recognized with certainty; no other infusoria were present. We believe, therefore, that in all these experiments, the meat had not been sufficiently heated to its centre, and that the experiment should be repeated in another manner.

§10. On the 18th of July, unfortunately the hottest season of the year, we again boiled meat in water, and while hot, corked it, and padded it over with cotton, as in §6 *d.*; a test fluid in an open flask beside it, showed, on the 22d of July, the odor of putrefaction, and on the 23d, a species of large infusoria could be recognized under the microscope, which we were not able to determine. They were globules or cells of the size of yeast globules, in constant voluntary motion, drawing themselves together like balls, and then stretching themselves out; we were, however, not able to perceive any further organization.

Upon the liquor that was under the cotton, we perceived a kind of fat skin, that covered the whole surface and gradually thickened. In the third week, the liquid acquired a reddish color; and upon being opened on the 15th of August, gave the odor of stinking fat, which, however, upon being warmed, was mixed with the odor of fresh broth.

We feel obliged to mention this experiment, because we do not consider ourselves justified in withholding it; not because we think any particular stress should be laid upon it; conclusions must not be drawn from a single experiment of this kind. Our idea is, that the meat in question, had not been boiled long enough, for the boiling had been stopped as soon as the liquid foamed up, so that probably all the serum in the interior of the meat was not coagulated. It is also very possible that from the high temperature at that time, the fat became rancid, which might have taken place even after boiling a long time. This can only be determined by further experiments; but even in this experiment, made during the hottest weather, putrefaction did not occur within 28 days.

§11. Although we believe that we have obtained from these experiments decidedly positive results, yet they should by no means be considered as concluded.

It appears then settled that there is a spontaneous decomposition of organic substances, as the putrefaction, of meat without water,—of the casein of milk—as well as the transformation of the sugar of milk in the milk into lactic acid, that requires for its commencement only the oxygen of the air, and that there are other phenomena of fermentation and putrefaction, which are improperly placed in the same category; viz: the fermentation of malt wort, and the putrefaction of meat under broth, which require for their commencement, beside the oxygen, some unknown admixture in the atmospheric air; which, according to Schwann's experiments, is destroyed by heat, and according to ours, removed by filtration through cotton. It will be a problem for future experiments, to divide into two classes those phenomena, which are now united under the general idea of fermentation and putrefaction. Our attention hereafter will be particularly devoted to certain simple organic combinations, viz: pure albumen, pure fibrin, casein free from fat, &c. &c.

§12. We have as yet only made use of cotton, as a means of filtration. It will be the object of future experiments to try a number of other substances for this purpose. We shall first use coal, sulphuret of lead, pumice stone, powdered glass, gypsum, &c. &c.

It is yet to be investigated, whether certain filtering substances will not remove the germs of one species of putrefaction and fermentation, permitting those of another to pass through, which in its turn may be removed by some other filtering medium; thus dividing the filtering substances into different classes.

Since there is still so much that remains undecided in an experimental way, we at present withhold all theoretical deductions from our researches.

The experiments mentioned above, will require from their nature, so long a lapse of time, that we do not think it right to withhold any longer from the public, the positive results already obtained. H. P.

*Manheim, Dec. 6th,* 1853. *Leibig's Annalen, Feb.* 1854.

